# Neutrophil-to-lymphocyte ratio predicts survival in European patients with hepatocellular carcinoma administered sorafenib

**DOI:** 10.18632/oncotarget.21528

**Published:** 2017-10-05

**Authors:** Alberto Lué, Maria Trinidad Serrano, Francisco Javier Bustamante, Mercedes Iñarrairaegui, Juan Ignacio Arenas, Milagros Testillano, Sara Lorente, Cristina Gil, Manuel de la Torre, Alexandra Gomez, Bruno Sangro

**Affiliations:** ^1^ Department of Gastroenterology, Hospital Clínico Universitario Lozano Blesa, 50009, Zaragoza, Spain; ^2^ Instituto de Investigación Sanitaria (IIS) Aragón, 50009, Zaragoza, Spain; ^3^ Department of Gastroenterology, Hospital Universitario Cruces, Plaza de Cruces, 48903, Barakaldo, Spain; ^4^ Liver Unit, Clinica Universidad de Navarra-IDISNA, 31008, Pamplona, Spain; ^5^ Centro de Investigacion Biomedica en Red de Enfermedades Hepaticas y Digestivas (CIBEREHD), 31008, Pamplona, Spain; ^6^ Department of Gastroenterology, Hospital Universitario Donostia, Begiristain Doktorea Pasealekua, 20014, San Sebastian, Spain

**Keywords:** neutrophil-to-lymphocyte ratio, hepatocellular carcinoma, sorafenib, overall survival

## Abstract

Neutrophil-to-lymphocyte ratio (NLR) is considered a prognostic factor in patients with hepatocellular carcinoma (HCC). Our aim is to investigate the prognostic significance of NLR in patients with HCC treated with sorafenib.

**Results:**

Median follow-up time was 7 months. Patients were mostly in the intermediate (27.3%) or advanced (72.7%) BCLC stages, 38.6% had vascular invasion and 27.5% extrahepatic disease. A large proportion (38.9%) had been previously treated with TACE. Liver function was preserved: 65.8% were classed as Child A. Median overall survival was 7.7 months (95% CI: 5.8–9.6). In univariate analysis, vascular invasion (*P* = 0.004), ECOG-PS ≥ 1 (*P* < 0.001), high bilirubin (*P* < 0.001), clinical ascites (*P* = 0.036), BCLC stage (*P* = 0.004), no previous TACE (*P* = 0.041) and NRL ≥ 2.3 (*P* = 0.005) were predictors of poor survival. Skin toxicity (*P* = 0.039) or hypertension (*P* = 0.033) during treatment were related to better survival. In multivariate analysis NLR ≥ 2.3 [HR 1.72 (95% CI: 1.03–2.71)], hyperbilirubinemia [HR 3.42 (95% CI: 1.87–6.25)] and ECOG-PS ≥ 1 [HR 1.97 (95% CI: 1.19–3.26)] were found as independent indicators of poor overall survival. Dermatologic adverse effects were an indicator of good overall survival [HR 0.59 (95% CI: 0.38–0.92)].

**Material and Methods:**

One hundred and fifty-four consecutive HCC patients treated with sorafenib in four different Spanish hospitals between August 2005 and October 2013 were analysed. Clinical, laboratory, and tumour features were obtained. Survival was calculated from the moment sorafenib treatment was initiated. Log-rank and Cox regression were used to analyse the ability of NLR to predict survival.

**Conclusions:**

NLR is an independent prognostic indicator for overall survival in HCC patients treated with sorafenib.

## INTRODUCTION

Hepatocellular carcinoma (HCC) is the fifth most common malignancy and the second leading cause of cancer-related deaths worldwide [1]. Liver transplantation, radiofrequency ablation, and surgical resection are considered potential curative treatments at the early stage of the disease [1, 2]. Locoregional treatments including transarterial chemoembolization (TACE) and radioembolization (TARE) are the mainstay of treatment for intermediate stage HCC, and the latter can be performed in the presence of macroscopic portal vein thrombosis [1–3]. In the advanced stage, sorafenib has been shown to improve survival in the first line of therapy [2, 4]. Recently regorafenib showed superiority over placebo in the second line and lenvatinib was non-inferior compared to sorafenib in a phase III trial in the same stage [5]. Patients in the intermediate stage with disease progression or not fit for locoregional therapy are treated with sorafenib based on the concept of treatment stage migration [6].

Although tumour stages define groups of patients with progressively worse prognosis, patients in the same stage may still have heterogeneous outcomes. For the intermediate stage, for instance, a subclassification into four groups has been recently proposed [7, 8], but we lack universally accepted prognostic factors that may allow stratification of patients across tumour stages [9, 10]. In the advanced stage some prognostic factors have been identified in the last years. Baseline AST could be helpful in determining which patients are most likely to benefit from sorafenib, especially inside Child B stage [11, 12]. Other authors reported that the development of adverse effect related to sorafenib treatment (dermatologic, diarrhoea and hypertension) has been associated with a better overall survival [13, 14]. Lastly in the GIDEON cohort, that includes more 3202 patients, the authors observed that patient of advanced Child stage have a poor overall survival [15]. Recently, the role of systemic inflammation in tumour progression has been investigated in different types of cancers. An indirect marker of a systemic inflammatory response is the neutrophil-to-lymphocyte ratio (NLR), which is defined as a ratio of the neutrophil to lymphocyte count in peripheral blood [16]. NLR has been evaluated as a prognostic factor in different malignancies including breast, ovarian, oesophageal, gastric, colorectal, kidney, and urothelial cancer [17–23]. In HCC, the impact of NLR on recurrence and survival has been evaluated in large cohorts of patients including those treated by surgical resection, transplantation, radiofrequency ablation, TACE, and TARE [16, 24–29]. In these studies, a high NLR predicted HCC recurrence and was associated with worse survival. The analysis of the tumour microenvironment in liver explants bearing HCC suggests that a high NLR is linked to an up-regulation of the inflammatory pathways that may confer a more aggressive tumour [30]. A clear cut-off value has not been established for NLR, and the values associated with a worse prognosis vary amongst different publications [31]. A recent review and meta-analysis suggests that NLR is a major prognostic factor for HCC patients and that it might be further incorporated into the prognostic model of HCC [32]. Data in sorafenib treated patients are scarce. A recent study including 56 patients observed that NLR represent a potential prognostic indicator in patients with advanced HCC treated with sorafenib. In this cohort a high NLR was associated with a lower progression free survival, but not with a significant change in overall survival [33]. The aim of our study is to investigate the prognostic significance of NLR in HCC patients treated with sorafenib.

## RESULTS

### Baseline characteristics

One hundred and fifty four patients with HCC that began therapy with sorafenib in the defined period were studied and their baseline characteristics are shown in Table 1. Briefly, most patients were men (79.9%) and Caucasian (95.7%). Mean age at the beginning of treatment with sorafenib was 63 (± 11) years. Most patients (88.4%) were cirrhotics and the most frequent aetiologies of liver disease were alcohol (39.4%) and hepatitis C virus infection (38.6%). Child-Pugh class at the time sorafenib treatment was initiated was A in 65.8% of patients and B in 34.2%. Less than one-fifth of patients had ascites (14.8%) and only 3.3% had had previous encephalopathy. With respect to staging, 27.3% of patients were in the intermediate stage (BCLC B) and 72.7% in the advanced stage (BCLC C) respectively. A significant number of patients (38.6%) had macroscopic vascular invasion and nearly one fourth (27.5%) had metastatic extrahepatic disease. An altered ECOG performance status was observed in 48 patients with 27.2% classified as grade 1 and 5.4% as grade 2. Median time between diagnosis and initiation of treatment with sorafenib was 4 months (range 1–12). A remarkable proportion of patients (38.9%) had received TACE prior to treatment with sorafenib and nearly one fifth (18.8%) had been treated with TARE. Globally 61% of patients had received at least one treatment before starting sorafenib. A significant number of patients reported any side effect (87.9%) during treatment with sorafenib. The most frequent adverse event was diarrhoea (46.8%), followed by dermatologic side effects (44%) and hypertension (15.6%).

**Table 1:** Baseline characteristics

Variable	
Gender	
Male	123 (79.9%)
Female	31 (20.1%)
Age (years)	63 ± 11 (28–86)
Ethnicity (*n* = 134)	
Caucasian	125 (95.4 %)
Others	6 (4.6%)
Cirrhosis (*n* = 130)	118 (90%)
Aetiology	
Alcohol	61 (39.4%)
HCV	59 (38.6%)
HBV	22 (14.4%)
Child Pugh (*n* = 146)	
Stage	
A	96 (65.8%)
B	50 (34.2%)
Score	6 ± 1 (5–9)
MELD	10 ± 3
Ascites	22 (14.8%)
Encephalopathy	5 (3.3%)
Albumin (g/dl)	3.6 ± 0.7
Hypoalbuminemia (≤ 3.5 g/dl)	47 (32.6%)
Creatinine (mg/dl)	0.9 ± 0.4
Bilirubin (mg/dl)	1.4 ± 0.8
Hyperbilirubinemia (≥ 2 mg/dl)	24 (15.9%)
Platelets (per microliter)	160.001 ± 93.361
INR	1.17 ± 0.31 (0.88–3.99)
BCLC Stage	
B	42 (27.3%)
C	112 (72.7%)
Vascular Invasion	59 (36.8%)
Extrahepatic metastases	42 (27.5%)
ECOG (*n* = 147)	
0	99 (67.3%)
1	40 (27.2%)
2	8 (5.4 %)
Alphafetoprotein (ng/ml)	142 (11–1.500)
Alphafetoprotein ≥ 400 ng/ml	56 (39.4%)
Previous treated	94 (61%)
Previous TACE treatment	60 (38.9%)
Previous TARE treatment	29 (18.8%)
AST (U/L)	63 (43–120)
AST ≥ 100 U/L	45 (30.6%)
Neutrophil (per microliter)	3,800 ± 2,200
Lymphocyte (per microliter)	1,400 ± 900
Neutrophil to Lymphocyte Ratio (NLR)	2.7 (1.8–4.1)
NLR ≥ 2.3	87 (57.6%)

### Survival analysis and neutrophil-to-lymphocyte ratio

Median follow-up was 7 months (IQR: 3–15 months). During this time, 117 deaths occurred, corresponding to 83.6% of the study population. Median overall survival was 7.7 months (95%CI: 5.8–9.6 months). Mean neutrophil count was 3,800 (± 2,200) per microliter, whereas mean lymphocyte count was 1,400 (± 900) per microliter. Median NLR was 2.7 (1.8–4.1). ROC analysis and Youden’s index were used to calculate an optimal cut-off level of 2.3. Sixty-four patients (42.4%) had a NLR value of less than 2.3.

**Figure 1:**
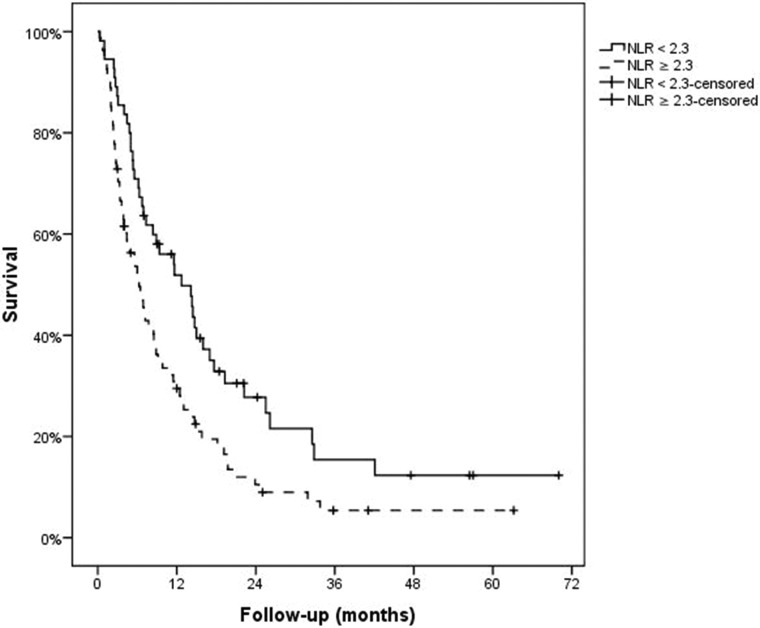
Overall survival according to neutrophil-to-lymphocyte ratio

Patients with NLR **≥** 2.3 had worse overall survival (6.3 months [95% CI: 4.3–8.3 months] vs. 12.7 months [95% CI: 7.2–18.2 months]; *P* = 0.005) Figure 1 shows the Kaplan Meier Overall survival curves according to neutrophil-to-lymphocyte ratio. A univariate analysis was performed to identify factors related to overall survival. An advanced BCLC C stage (*P* = 0.005), clinical ascites (*P* = 0.036), vascular invasion (*P* = 0.004), hyperbilirubinemia (*P* < 0.001), absence of previous TACE (*P* = 0.041), and ECOG performance status ≥ 1 (*P* < 0.001) were statistically associated with poor survival. Skin toxicity (*P* = 0.039) and hypertension (*P* = 0.033) during treatment with sorafenib were statistically associated with better survival. We did not observe an association between survival and Child score (*P* = 0.193), hypoalbuminemia (*P* = 0.264), high alphafetoprotein (≥ 400 ng/ml) (*P* = 0.158), high GOT/AST levels (**≥** 100 U/L) (*P* =0.059), extrahepatic disease (*P* = 0.632), hepatic encephalopathy (*P* = 0.903), previous RE (*P* = 0.956), or any treatment prior to sorafenib (*P* = 0.068). Univariate analysis results for overall survival are outlined in Table 2. A multivariate model was built including those variables that were statistically significant in the multivariate analysis: high NLR, clinical ascites, vascular invasion, hyperbilirubinemia, absence of previous TACE, ECOG performance status ≥ 1, dermatologic side effects and hypertension. BCLC C stage was not included to avoid redundance since 2 of its 3 components (vascular invasion and altered ECOG status were included). In this model a NLR ≥ 2.3 was confirmed as an independent indicator of poor overall survival together with ECOG performance status ≥ 1 and hyperbilirubinemia. Dermatologic side effects were confirmed as independent indicator of better overall survival. Results of multivariate analysis are summarized in Table 3.

**Table 2:** Overall survival-univariate analysis

Variable	Median survival (months) (95% CI)	*P*
NLR ≥ 2.3		**0.005**
Yes	6.28 (4.29–8.27)	
No	12.72 (7.24–18.21)	
AFP ≥ 400 ng/ml		0.158
Yes	6.31 (4.43–8.19)	
No	9.37 (5.95–12.79)	
AST ≥ 100 U/L		0.059
Yes	4.44 (1.07–7.80)	
No	8.51 (4.96–12.07)	
Any adverse effects		0.519
Yes	10.88 (7.78–13.99)	
No	4 (0–8.47)	
Dermatologic manifestations		**0.039**
Yes	14.30 (10.72–17.88)	
No	6.18 (3.22–9.14)	
Diarrhoea		0.273
Yes	11.64 (5.18–13.10)	
No	6.71 (3.83–9.58)	
Hypertension		**0.033**
Yes	6.71 (3.83–9.58)	
No	7.2 (4.55–9.85)	
BCLC Stage		**0.006**
B	15 (13.35–16.65)	
C	6.48 (4.75–8.20)	
Presence of vascular invasion		**0.004**
Yes	5.65 (3.96–7.35)	
No	11.57 (6.57–16.57)	
ECOG ≥ 1		**< 0.001**
Yes	5 (2.67–7.33)	
No	11.80 (6.60–17)	
Extrahepatic dissemination		0.632
Yes	6.94 (2.93–10.95)	
No	8.51 (6.03–10.99)	
Child Stage		0.193
A	8.38 (5.82–10.95)	
B	6.74 (2.21–11.27)	
Hyperbilirubinemia (≥ 2 mg/dl)		**< 0.001**
Yes	3.39 (0.63–9.64)	
No	8.91 (5.27–12.55)	
Hypoalbuminemia (≤ 3, 5 g/dl)		0.264
Yes	7 (3.82–10.18)	
No	8.48 (5.27–11.69)	
Clinical Ascites		**0.036**
Yes	4.83 (1.75–7.92)	
No	8.51 (4.78–12.25)	
Encephalopathy		0.903
Yes	8.91 (0–20.93)	
No	7.33 (5.26–9.40)	
Previous treated		0.068
Yes	10.88 (7.59–14.17)	
No	5.33 (3.43–7.22)	
Previous TACE		**0.041**
Yes	11.64 (7.68–7.75)	
No	6.18 (4.61–7.47)	
Previous TARE		0.956
Yes	6.71 (4.70–8.71)	
No	8.38 (6.15–10.62)	

**Table 3:** Overall Survival-Multivariate Analysis (adjusted for NLR ≥ 2.3, ECOG-PS ≥ 1, dermatological adverse events, hyperbilirrubinemia, hypertension, presence of vascular invasion, clinical ascites and previous TACE)

Variable	*P*	HR (95% CI)
NLR ≥ 2.3	**0.019**	1.72 (1.09–2.71)
ECOG-PS ≥ 1	**0.008**	1.97 (1.19–3.26)
Dermatological adverse events	**0.019**	0.59 (0.38–0.92)
Hyperbilirrubinemia (≥ 2 mg/dl)	**< 0.001**	3.42 (1.87–6.25)

## DISCUSSION

The interest in systemic inflammation as a poor prognostic marker in patients with cancer is growing [17–23]. An increased systemic inflammatory response may be associated with a poor performance status [34, 35]. However, in patients with HCC, the identification of an altered performance status due to cancer can be a challenge because the symptoms related to the underlying cirrhosis [34] and subclinical inflammation may worsen patient prognosis. Laboratory tests used to identify systemic inflammatory markers such as serum levels of C-reactive protein or albumin may overcome these two problems [28] however, these parameters might be altered in cirrhotic patients with or without tumour disease, therefore their utility is questionable. NLR is one of the easiest and most reliable indexes used to detect a systemic inflammatory response.

NLR has been previously studied in patients with HCC; however, mostly in the context of surgery, transplantation, or TACE [24–29]. Recently the prognostic value of NLR has been also evaluated in a group of patient included in a phase II randomized trial evaluating tivantinib vs. placebo. In this study the authors observed that a baseline high NLR (greater than 3) is an independent prognostic biomarker in patients with HCC who are candidate for second-line treatments. An important finding was that for overall survival, no interaction was detected between NLR status and treatment [36]. These results confirm that NLR have a prognostic value regardless the treatment applied.

Our study is one of the largest performed in a European population and one of the first to demonstrate the prognostic value on overall survival of high NLR values in European patients with advanced HCC treated with sorafenib. A single study on a small sample of Chinese patients with extremely high NLR values found similar results [37]. Another Chinese cohort of 205 patients those with low NLR presented lower CLIP score and higher 6-month survival rate (56.1 vs 25.9%) compared with patients with high NLR level (greater than 2.43). Moreover low NLR level was associated with favorable prognostic factors such as lower α-fetoprotein, alkaline phosphatase, and total bilirubin, as well as decreased incidence of ascites, portal vein thrombosis, and metastasis [38]. Nevertheless, the characteristics of the populations receiving sorafenib in European and Eastern countries are not comparable as illustrated in the wide difference in overall survival reported in the pivotal trials [4, 39]. Another recent study including 56 patients with advanced hepatocellular carcinoma treated with sorafenib observed that a high NLR (greater that 3) was associated with a lower progression free survival (*P* = 0.049), but not with a lower overall survival in the multivariate analysis (*P* = 0.062), probably because the number of patients included [33].

Similar results were observed in a Brazilian cohort of 105 patients treated with sorafenib. In this study the authors observed that a high NLR (greater than 3.5) was associated with a worse prognosis [40]. Our study confirms and complements the results of these studies.

Recently a large cohort of 442 patients treated with sorafenib among three different countries demonstrated that the integration of inflammatory markers, clinical features and treatment related side-effects could help stratifying patients according to survival. Particularly the authors observed that previously-treated HCC, Cancer of Liver Italian Program (CLIP) score, baseline red cell distribution width and NLR were significant independent risks for shorter survival, whilst sorafenib-related diarrhoea was associated with prolonged survival. Finally the authors purpose a novel prognostic index combining CLIP score with inflammatory marker and treatment-related side-effects has good accuracy for predicting survival in patients with advanced HCC treated with sorafenib [41]. Our data support the results and the hypothesis of this study: clinical features, inflammatory markers and treatment related adverse are independent predictors of survival in advanced HCC patient treated with sorafenib.

In our study development of adverse effect related to sorafenib could help to identify patients with a better overall survival, as previously described [13, 14]. Particulary development of dermatologic adverse effects related to sorafenib is an independent predictor of good overall survival in our cohort.

The characteristics of our cohort are those expected in a population of patients treated with sorafenib [8]. The NLR cut-off level in our study is consistent with other publications in which ROC curves were used to identify such value [28, 42]. The higher cut-off values observed in other studies, especially those involving Asian populations, are in line with the differing patient characteristics mentioned above [30, 37, 43]. Indeed, a recent meta-analysis that included 15 studies and investigated the prognostic impact of NLR on survival with different HCC treatments, was unable to establish a clear cut-off value [43]. Results from another recent review and meta-analysis support the incorporation of NLR into the prognostic model of HCC, but did not set a clear cut-off [32].

In our analysis, NLR emerged as a predictor of overall survival. Overall, survival of patients with a NLR ≥ 2.3 was significantly reduced compared to patients with a lower NLR (6.29 vs. 12.72 months). Not surprisingly, factors most strongly associated with survival were an advanced BCLC stage and particularly one of their components: altered performance status.

We did not observe that alpha-fetoprotein levels were associated with poor survival, probably reflecting that this variable is a better predictor when used in a dynamic way [8, 44].

Those patients that received sorafenib after having progressed to TACE had a better outcome. The likely cause of this observation is that those patients that are still eligible for sorafenib after TACE failure represent a select group of tumours with a less aggressive behaviour. There may be also a confounding effect by indication bias in this group (Reference). Previous TACE treatment could select a group of patients with better survival because of their clinical characteristics rather than TACE treatment itself. This hypothesis would be supported by the fact that prior TACE was not independently associated with mortality in the multivariate analysis.

In conclusion, our results indicate that a high NLR is a strong and easily available predictor of overall survival in patients with intermediate to advanced HCC treated with sorafenib. Along with other studies involving patients at different stages and those administered different therapies, our results show that NLR is a simple, objective, inexpensive, and readily available biochemical marker that may be useful in refining the prognosis of individual patients with HCC. Quite importantly, NLR could help stratify these patients in clinical trials of systemic therapies with overall survival as the main endpoint.

## MATERIALS AND METHODS

### Patients and variables

All consecutive patients with HCC that began therapy with sorafenib between August 2005 and October 2013 in four different Spanish hospitals were considered for analysis. The diagnosis of HCC was based on non-invasive criteria [2, 45] or histology. Tumour staging was determined according to the Barcelona clinic liver cancer (BCLC) classification [2, 45]. Patients with previous liver transplantation, stage A or D of BCLC classification and stage C of Child classification were excluded from analysis. The data were collected from clinical records in an anonymous registry and were retrospectively analysed. The collection of data in the registry has the approval of the local ethics committee (CEICA, Comité Ético de Investigación Clinica de Aragón, Zaragoza, Spain).

Baseline patient characteristics were obtained at the beginning of sorafenib treatment. Demographic characteristics included age, gender, and ethnic group. Hepatic disease features included the presence of cirrhosis; aetiology of liver disease; presence and degree of ascites and encephalopathy; serum levels of albumin, bilirubin, AST/GOT, creatinine, and alpha-fetoprotein; platelet count; INR; and prothrombin activity as well as composite scores such as Child-Pugh stage and MELD score. Tumour characteristics included BCLC stage, number of nodules, size of the largest nodule, presence of vascular invasion and metastases, ECOG performance status, and previous loco-regional treatment. BCLC and Child stages were analysed as qualitative variables. NLR was calculated as the ratio of neutrophil count to lymphocyte count in peripheral blood measured just prior to the beginning of sorafenib treatment. Development of adverse events (dermatologic manifestations, hypertension or diarrhoea) during treatment with sorefenib was collected. Overall survival was calculated from the date Sorafenib was initiated to the date of death or last follow-up visit.

### Statistical analysis

Qualitative variables are expressed as a percentage, whereas continuous variables are expressed as mean (± standard deviation) if they had a normal distribution and as median (interquartile range) if otherwise. Survival plots were calculated using the Kaplan-Meier method. The Log-Rank method was used for univariate analysis and the Cox-regression model was used for multivariate analysis. The likelihood ratios forward stepwise method was used for the multivariate Cox proportional analysis. The NLR cut-off level was calculated using the receiver operator curve (ROC) analysis and Youden’s index with death as test variable [28, 42]. For AFP (400 ng/ml) and AST/GOT (100 U/L) cut off levels we used those previously described [11, 12, 46]. A *p*-value of less than 0.05 was considered statistically significant. SPSS version 22 (SPSS Inc., Chicago, IL, USA, Universidad de Zaragoza) was used for all statistical analyses.

## Abbreviations

BCLC: Barcelona clinic liver cancer
HCC: hepatocellular carcinoma
NLR: neutrophil-to-lymphocyte ratio
TACE: transcatheter arterial embolization
TARE: transarterial radioembolization
